# Conserved metabolic enzymes as vaccine antigens for giardiasis

**DOI:** 10.1371/journal.pntd.0010323

**Published:** 2022-04-25

**Authors:** Sozaburo Ihara, Yukiko Miyamoto, Christine H. Y. Le, Vivien N. Tran, Elaine M. Hanson, Marvin Fischer, Kurt Hanevik, Lars Eckmann

**Affiliations:** 1 Department of Medicine, University of California San Diego, La Jolla, California; 2 Division of Gastroenterology, The Institute for Adult Diseases, Asahi Life Foundation, Tokyo, Japan; 3 Department of Clinical Science, University of Bergen, Bergen, Norway; Universidade Federal de São Paulo, BRAZIL

## Abstract

*Giardia lamblia* is a leading protozoal cause of diarrheal disease worldwide. Infection is associated with abdominal pain, malabsorption and weight loss, and protracted post-infectious syndromes. A human vaccine is not available against *G*. *lamblia*. Prior studies with human and murine immune sera have identified several parasite antigens, including surface proteins and metabolic enzymes with intracellular functions. While surface proteins have demonstrated vaccine potential, they can exhibit significant variation between *G*. *lamblia* strains. By comparison, metabolic enzymes show greater conservation but their vaccine potential has not been established. To determine whether such proteins can serve as vaccine candidates, we focused on two enzymes, α-enolase (ENO) and ornithine carbamoyl transferase (OCT), which are involved in glycolysis and arginine metabolism, respectively. We show in a cohort of patients with confirmed giardiasis that both enzymes are immunogenic. Intranasal immunization with either enzyme antigen in mice induced strong systemic IgG1 and IgG2b responses and modest mucosal IgA responses, and a marked 100- to 1,000-fold reduction in peak trophozoite load upon oral *G*. *lamblia* challenge. ENO immunization also reduced the extent and duration of cyst excretion. Examination of 44 cytokines showed only minimal intestinal changes in immunized mice, although a modest increase of CCL22 was observed in ENO-immunized mice. Spectral flow cytometry revealed increased numbers and activation state of CD4 T cells in the small intestine and an increase in α4β7-expressing CD4 T cells in mesenteric lymph nodes of ENO-immunized mice. Consistent with a key role of CD4 T cells, immunization of CD4-deficient and Rag-2 deficient mice failed to induce protection, whereas mice lacking IgA were fully protected by immunization, indicating that immunity was CD4 T cell-dependent but IgA-independent. These results demonstrate that conserved metabolic enzymes can be effective vaccine antigens for protection against *G*. *lamblia* infection, thereby expanding the repertoire of candidate antigens beyond primary surface proteins.

## Author summary

*Giardia lamblia* is a leading parasitic cause of diarrheal disease worldwide. Symptomatic infection is associated with abdominal pain, malabsorption and weight loss, and can lead to protracted post-infectious syndromes. Despite its medical importance, a human vaccine has not been developed against the pathogen. Here we show that two highly conserved enzymes with metabolic functions in the cytoplasm of the parasite can be used as antigens for effective vaccines against *G*. *lamblia*, thereby significantly expanding the repertoire of candidate antigens against this clinically important infection beyond primary surface proteins.

## Introduction

*Giardia lamblia* causes one of the most common protozoal infections of the human intestinal tract with an estimated 280 million cases every year [1]. It is an important worldwide cause of diarrheal disease and a leading factor in delayed childhood development in resource-limited settings [2]. Symptomatic infection is characterized by diarrhea, abdominal pain, malabsorption and weight loss, and can lead to protracted post-infectious syndromes [3]. Clearance occurs spontaneously over weeks and months in most cases, but recurrent and chronic infections are common in endemic regions [4]. The reasons for the wide variation in clinical symptoms and natural history are not well understood, but microbial diversity and differences in host immune responses are likely to play a role [1,5,6].

Treatment of giardiasis is most commonly with 5-nitroimidazole compounds, particularly metronidazole, although other anti-giardial drugs are available. However, treatment failures occur (in up to 40% of cases in some studies) and resistance to all anti-giardial drugs has been documented [7,8]. Despite its medical importance, no human vaccine is available against giardiasis. A crude veterinary vaccine (GiardiaVax), composed of total cell lysates of a mixture of sheep, dog and human isolates, was developed and has been shown to reduce symptoms and duration of cyst output in cats and dogs [9], and even be effective post-exposure [10], but its production has been discontinued and it is no longer commercially available. Attenuated forms of *G*. *lamblia* that might be used as live vaccines have not been reported; such forms are not likely to establish infection in the host and would thus probably fail to activate mucosal immunity.

Multiple *G*. *lamblia* proteins are recognized by immune sera from infected humans and animals [11–14]. Among the best-characterized antigens are variant-specific proteins (VSPs), whose functions remain poorly understood [15]. Some 200 different VSP genes are in the *G*. *lamblia* genome [16,17], but only one is expressed per trophozoite [18]. Switches in VSP expression are common [19], confounding development of VSP-specific immune protection. VSP expression is regulated by RNA interference, and its silencing allows concurrent expression of multiple VSPs in individual trophozoites and has been proposed as a basis for a vaccine [20–22]. However, the human-pathogenic Assemblage A and B isolates have completely different VSP repertoires with >400 antigens altogether [16,17,23,24], greatly complicating development of tractable multi-antigen vaccines.

Several non-VSP antigens of *G*. *lamblia* have been identified, including giardins, α-enolase (ENO), ornithine carbamoyl transferase (OCT), and arginine deiminase [13,14,25,26], which are conserved and do not undergo antigenic variation. Several of these proteins, including α1- and α11-giardin, uridine phosphorylase-like protein-1, and protein 21.1, are expressed on the surface of trophozoites and can confer protection upon immunization in murine models [26]. In contrast, proteins that have primary enzymatic functions, such as ENO and OCT, have so far not provided immune protection in murine models when expressed in live vaccine vectors [27]. Nonetheless, such proteins are generally well conserved between different *G*. *lamblia* strains, making them potentially attractive vaccine antigens if they could be shown to be immunogenic and protective. Therefore, in this study, we sought to determine the vaccine antigen potential of two of these proteins, ENO and OCT, as representative metabolic enzymes of *G*. *lamblia*.

## Methods

### Ethics statement

All participants provided written informed consent and the study was approved by the Regional Committees for Medical and Health Research Ethics (REC WEST, Norway) number 2016/1632. Animal care and use for this study was approved by the University of California, San Diego Institutional Animal Care and Use Committee under protocol number S00205 and United States Public Health Service assurance numbers A3033-1 and D16-00020. Animal use adhered to the guidelines in the most recent edition of the Guide for the Care and Use of Laboratory Animals of the National Research Council of the United States National Academies and the Guidelines on Euthanasia by the American Veterinary Medical Association.

### Mice

C57BL/6 mice, BALB/cJ mice, and CD4 knock-out (KO) and Rag-2 KO mice on a C57BL/6 background were obtained from the Jackson Laboratory. IgA KO mice were described before [28]. Mice were ≥6 weeks old for experiments. No significant differences were observed between males and females, so results were combined.

### *G*. *lamblia* isolates and cultivation

*G*. *lamblia* strains GS/M clone H7 (GS/M; ATCC 50581) and WB/C6 (WB, ATCC 50803) were used [29]. Parasites were routinely grown in modified TYI-S-33 medium to no more than 80% confluence [30].

### Production of recombinant proteins

Sequences of ENO and OCT were obtained from the genome of *G*. *lamblia* GS/M [17]. Sequence comparison between different *G*. *lamblia* assemblages and humans were done with BlastP (National Center for Biotechnology Information). Coding DNAs were generated by total gene synthesis with codon optimization for *Escherichia coli* (GenScript) and inserted into NdeI-XhoI cloning sites of the bacterial expression vector pET-15b (Millipore Sigma). Vectors were transformed into T7 Express lysY/Iq Competent *E*. *coli* (NEB) per the manufacturer’s protocol. Cells were grown at 37°C in LB medium with carbenicillin until they reached an optical density at 600 nm (OD600) of 0.4 to 0.6, and protein expression was induced by addition of 1 mM isopropyl-β-d-thiogalactopyranoside (IPTG) for 2 to 3 h. Recombinant proteins were purified from bacterial lysates using HisPur Ni-nitrilotriacetic acid (NTA) resin (Thermo Scientific). Production and purification of α1-giardin and uridine phosphorylase-like protein-1 were described before [26,27]. Recombinant proteins were stored at a concentration of >1 mg/ml at 4°C in a buffer of 300 mM NaCl, 25 mM HEPES, 100 mM arginine, 20 mM imidazole, 10% glycerol, 0.1% Tween 20, and protease inhibitors (Complete Mini-EDTA, Roche). Purity of the soluble recombinant proteins was analyzed by SDS-PAGE and Coomassie Blue staining.

### Human serum samples and multiplex beads immunoassays

Serum samples from 31 Norwegian patients with laboratory-confirmed giardiasis were collected [31]. The patients were all adults and most had acquired infection by visiting other countries including Africa (n  =  11), Asia (n  =  6), the Americas (n  =  4), and Europe (n  =  7), and three were of uncertain origin. Control sera were obtained from 19 healthy presumed-unexposed adults. Patients and controls were matched for age (means and standard deviations of 42.3 ± 20.4 and 39.6 ± 16.8 years, respectively) and sex (45% and 37% female, respectively). The Regional Committee for Medical Research Ethics of Western Norway approved the study.

For determination of specific antibodies in serum, recombinant *Giardia* antigens or glutathione S-transferase (GST) as a control protein was separately coupled to 4-μm Cyto-Plex polystyrene beads with different levels of internal fluorescence (Thermo Fisher Scientific, Waltham, MA) using sodium N-hydroxysulfosuccinimide and N-(3-dimethylaminopropyl)-N’-ethylcarbodiimide HCl cross-linking chemistry. Bead density was determined by microscopic counting. Wells of a MultiScreen HTS HV filter plate were prewetted with 200 μl of assay buffer (PBS containing 1% BSA and 0.05% Tween 20) and 10^4^ beads per well of each protein-labeled type were added. Diluted human serum (1:50 in assay buffer) was added (100 μl/well), and plates were incubated for 1 h at room temperature. Subsequently, wells were washed using assay buffer and Alexa Fluor 555-labeled goat anti-human IgG(H+L) antibody (Jackson Immunoresearch, Ely, UK) or Alexa Fluor 488-labeled AffiniPure goat anti-human IgA was added to the wells (1:500 or 1:400 dilution, respectively, in 200 μl assay buffer). Samples were incubated for 1 h at room temperature. As positive controls, wells incubated with Alexa Fluor 488-labeled mouse anti-His antibody (1:100; Novus Biologicals, Abingdon, UK) were used, as the recombinant antigens carried the His epitope tag. As negative control we used GST-tagged beads. Following incubation, beads were washed twice in buffer (PBS containing 1% BSA), resuspended and transferred to a round-bottom microplate for analysis in an LSRFortessa flow cytometer (BD Biosciences, San Jose, CA). Quantification of IgG and IgA responses was performed by determining the median fluorescence intensity (MFI) for each protein-coupled bead population using FlowJo software, version 10.4.2 (FlowJo LLC, Ashland, OR).

### Antigen immunizations

For intranasal immunizations, mice were placed under anesthesia with ketamine and xylazine, and a small volume (6 μl) of a mixture of a purified antigen (20–40 μg) and cholera toxin (1 μg) was instilled into one of the nares. The antigens are detailed in the respective figures for specific experiments. Only one antigen was administered at a time. Single antigens were given every two weeks, while combinations of antigens were tested by consecutive weekly administrations of each antigen. Each antigen was administered three times. Endotoxin contamination in the inoculation mixtures was <0.8 EU/dose as determined with Limulus Amebocyte Lysate assay (ToxinSensor Chromogenic LAL Endotoxin Assay Kit, Genscript). As controls, cholera toxin was either given alone without antigen, or mice were used naïve without any further manipulations. Mice were left recumbent for at least 1 min after intranasal administration and then allowed to recover in their cages.

### *G*. *lamblia* infections in mice

Infection challenges were done at least two weeks after the last immunization. Mice were treated for a week before and throughout the infection with an antibiotic cocktail (1.4 mg/ml neomycin, 1 mg/ml ampicillin, and 1 mg/ml vancomycin in the drinking water). *G*. *lamblia* trophozoites were grown to mid-logarithmic phase and administered in growth medium by oral gavage to mice (10^6^ trophozoites of strains GS/M or WB in a 200-μl volume) [26,32]. Mice were euthanized at different times after infection by controlled CO_2_ inhalation and the small intestine was removed. The intestine was opened longitudinally, placed into 2 ml of PBS, and cooled on ice for 10 min. After vigorous shaking of samples, live trophozoites in the supernatants were enumerated in a hemocytometer. Twenty 1 mm^2^ fields (containing 0.1 μL each) were counted for each sample, yielding a detection threshold of one trophozoite per 2 μL of sample, or 10^3^ trophozoites per small intestine. For analysis of fecal cyst output, fecal pellets were collected after placing mice into a clean cage without bedding for a 1–2 h period. Samples were weighed (~20–50 mg/mouse) and fixed in 10% formalin for 24 h at 4°C. After fixation, samples were homogenized, washed twice with PBS by centrifugation and resuspension, and stained for 30 mins at room temperature with a 1:100 dilution of FITC-labeled polyclonal goat anti-*Giardia* (LSBio) in PBS with 1% BSA. After three washes with PBS, samples were resuspended in 200 μl PBS and cysts were manually counted in a hemocytometer using a fluorescence microscope. All animal studies were reviewed and approved by the University of California San Diego Institutional Animal Care and Use Committee.

### Antigen-specific antibody assays

To determine antigen-specific antibody levels in intestinal mucosal secretions, the entire small intestine was removed, opened lengthwise in 2 ml of PBS with a protease inhibitor cocktail (Complete Mini-EDTA, Roche, plus 1 μM E-64, Sigma) and shaken for 5 s. After centrifugation (14,000 rpm, 5 min, 4°C), the supernatants were collected and stored at -20°C until analysis. For assays of antigen-specific antibody levels in blood, plasma was collected by tail vein bleeding into PBS containing 5 mM EDTA and subsequent centrifugation. Levels of antibodies specific for the respective immunizing antigens were assayed by ELISA [14]. Microtiter plates (Immulon 4 HBX; Thermo Electron) were coated by overnight incubation of 50 μl/well of a 5 μg/ml solution of purified antigen in PBS at 4°C. Plates were blocked with 5% dried non-fat milk in PBS for 2 h at room temperature. Plates were incubated with serial dilutions of plasma or mucosal washes (diluted in PBS containing 1% BSA) for 1 h at room temperature, washed with PBS, and further incubated with HRP-labeled goat anti-mouse IgA, IgM, IgG, IgG1, IgG2a, IgG2b, or IgG3 (Southern Biotech; 1/1,000 diluted in 1% goat serum in PBS) for 1 h at room temperature. After washing, bound HRP was detected with tetramethylbenzidine/H_2_O_2_ in 0.1 M sodium acetate buffer (pH 6.0). Reactions were stopped with 1.2 M sulfuric acid, and absorbance was read at 450 nm and, for background subtraction, at 750 nm.

### Tissue cytokine analysis

Small (~5 mm) pieces of small intestine were homogenized for 30 s on ice with a disposable mini-pestle (Pellet Pestle Cordless Motor, Fisher Scientific) in 1.5 ml microfuge tubes using RIPA extraction buffer (Cell Signaling Technology, Cat No 9806) and a cocktail of protease inhibitors (HALT, Thermo Scientific, Cat No 78429). After centrifugation (20,000 x g, 4°C, 15 min) to remove tissue debris, supernatants were recovered and measured for protein content (BCA Protein Assay, Fisher Scientific). Samples were diluted to 1 mg/ml protein with extraction buffer, and submitted for multiplex cytokine analysis (Mouse Cytokine/Chemokine 44-Plex Discovery Assay Array, Eve Technologies).

### Spectral flow cytometry

The small intestine was removed, opened lengthwise, cut into 1–2 cm pieces, and rinsed with cold HBSS to remove luminal content and detach any attached *G*. *lamblia* trophozoites for subsequent counting. Gut pieces were incubated in 1 mM DTT in PBS for 15 min at room temperature with gentle rocking to remove mucus, followed by further incubation for 30 min in 2 mM EDTA in PBS with gently rocking to remove the epithelial layer. The remaining gut pieces were incubated in tissue digestion buffer containing 1.5 mg/ml collagenase (Sigma, Cat No C2139), 0.5 mg/ml dispase II (Sigma, Cat No D4693) and 0.05 mg/ml DNase (Roche, Cat No 10104159001) in RPMI medium for 30 min at 37°C with gentle rocking (150 rpm/min). Samples were forcefully pipetted ten times to dislodge cells and filtered through a 70 μm filter to remove tissue debris and large cell clumps. The resulting single-cell suspensions were washed twice with cold PBS and resuspended at 10^7^ cells/ml in PBS. Aliquots of 2 x 10^6^ cells in 200 μL were stained with the viability dye Zombie NIR (BioLegend, Cat No 423105), followed by incubation with an Fc blocking solution (TruSTain FcX, BioLegend, Cat No 101320). After washing in PBS, cells were stained for 30 min on ice with an antibody master mixture in PBS with 4% fetal bovine serum. The master mixture contained the following fluorochrome-labeled monoclonal antibodies: FITC-Anti CD45 (clone 30-F11, BioLegend, Cat No 103108), PE/Dazzle-Anti CD3ε (clone 145-2C11, BioLegend, Cat No 100348), BV570-Anti CD4 (clone RM4-5, BioLegend, Cat No 100541), PE-Cy5-Anti CD8 (clone 53–6.7, BioLegend, Cat No 100710), BV510-Anti B220 (clone RA3-6B2, BioLegend, Cat No 103248), eFluor 450-Anti CD11b (M1/70, Thermo/Invitrogen, Cat No 48-0112-82), BV785-Anti CD11c (clone N418, BioLegend, Cat No 117336), BV480-Anti CD49d/integrin α4 (clone R1-2, BD Biosciences, Cat No 746526), AF 647-Anti CD103/integrin αE (clone 2E7, BioLegend, Cat No 121410), BV421-Anti integrin β7 (clone FIB504, BD Biosciences, Cat No 564283), AF 532-Anti MHC Class II (clone M5/114.15.2, Thermo/Invitrogen, Cat No 58-5321-82), and BV750-Anti CD44 (clone IM7, BD Biosciences, Cat No 747255). After staining, cells were washed and fixed with paraformaldehyde (BD Stabilizing Fixative, BD Bioscience) for 30 min on ice, followed by washing and storage in 4% FCS in PBS at 4°C until analysis. Stained samples were analyzed on a spectral flow cytometer (Aurora Cytometer, Cytek Biosciences) and data were analyzed with FloJo v10 (BD Biosciences). Viable single cells were identified among the total events by forward scatter-area and side scatter-area characteristics and negative staining for the Zombie NIR viability dye.

### Statistical analysis

An unpaired t test, Mann-Whitney U test, or Kruskal-Wallis test with Bonferroni correction and the appropriate post hoc tests were used to compare results between animal groups as appropriate. Data are presented as mean ± standard error (SE) or as individual data points and geometric means. For the human samples, differences in specific antibody responses between individuals in the exposed and presumed-unexposed group were analyzed by a Mann-Whitney U test using SPSS Statistics, version 24. Differences with P values of <0.05 were considered statistically significant for all experiments.

## Results

### Production of recombinant ENO and OCT

To confirm the degree of ENO and OCT conservation across different *G*. *lamblia* assemblages, we performed sequence analysis for the genes in the two divergent strains, WB (assemblage A) and GS/M (assemblage B). ENO has 95% amino acid sequence identity and 98% similarity between WB and GS/M. Similarly, OCT exhibits 97% amino acid sequence identity and 98% similarity between the two strains. In contrast, ENO has only 51% amino acid sequence identity with the most closely related human gene, neuron-specific enolase, and OCT only 31% identity with its human counterpart. These data indicate that the two enzymes are highly conserved among *G*. *lamblia* strains, yet substantially different from any human proteins, making them potential vaccine candidates.

**Fig 1:**
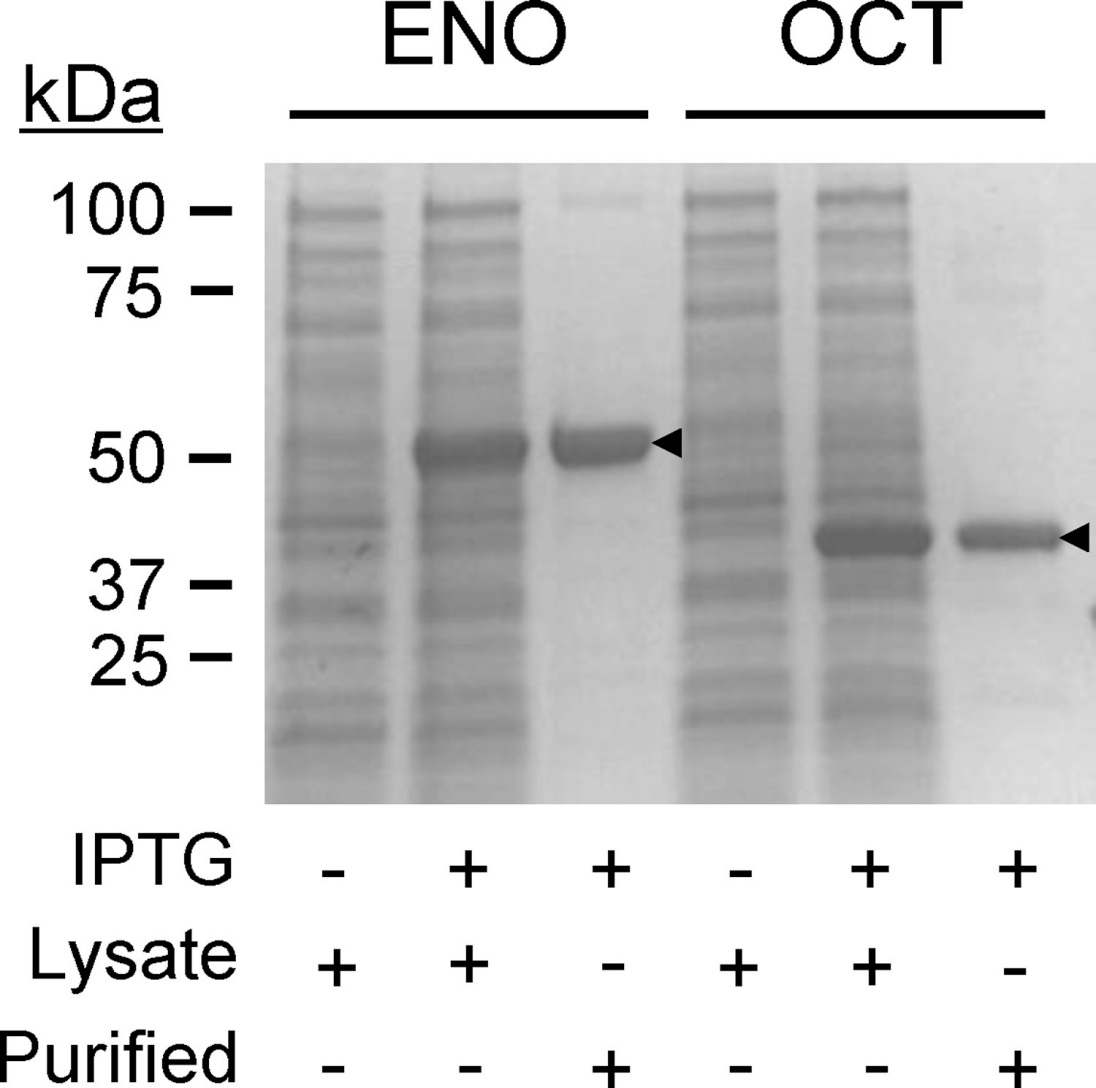
Production and purification of recombinant ENO and OCT. Recombinant forms of α-enolase (ENO) and ornithine carbamoyl transferase (OCT) were produced in *E*. *coli* after induction with isopropyl-β-d-thiogalactopyranoside (IPTG), purified by nickel affinity chromatography, and visualized by SDS-PAGE and Coomassie Blue staining. Arrow heads depict the recombinant proteins.

To conduct functional studies, we produced ENO or OCT as recombinant His-tagged proteins in *Escherichia coli* and purified them from bacterial lysates by Ni-NTA affinity chromatography (Fig 1). Estimated purity was 95% for ENO and 96% for OCT, as determined by Coomassie Blue staining of the proteins fractionated by SDS-PAGE (Fig 1). Endotoxin contamination of the antigen preparations was low (<30 EU/mg protein).

### Immunogenicity of ENO and OCT in human giardiasis

**Fig 2:**
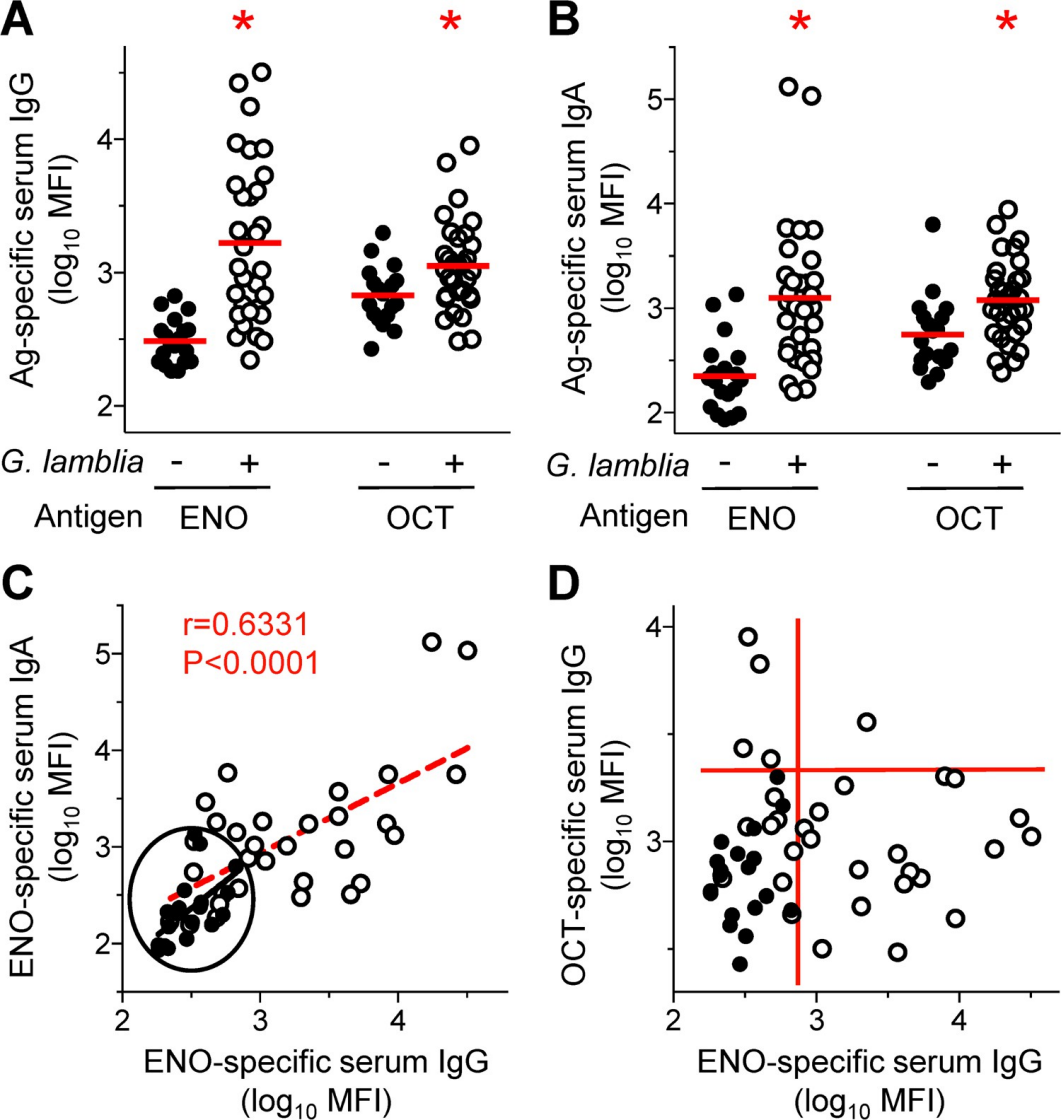
Immunogenicity of ENO and OCT in humans. A,B. Sera from *G*. *lamblia*-infected patients (circled dots) and age-matched, presumed-unexposed controls (black dots) were tested for levels of IgG (A) and IgA (B) against the indicated antigens (Ag) by multiplex bead immunoassays. Each data point represents one individual, red bars show the geometric means; *P<0.05 vs controls as determined by Mann-Whitney U test. C. Correlation of ENO-specific IgG and IgA levels. The red dashed line represents the linear correlation curve. The black oval encloses all control values. D. Correlation of levels of IgG specific for ENO and OCT. The red lines outline the different groups with low or high antibody levels against the two antigens, with the lower left quadrant including all controls.

To investigate the immunogenicity of ENO and OCT in humans, we used a cohort of 31 patients with laboratory-confirmed giardiasis [31] and 19 healthy, age- and sex-matched presumed-unexposed individuals as controls. Levels of antigen-specific IgG and IgA were determined in serum by multiplex bead immunoassay, using recombinant ENO and OCT as antigens. Prior *G*. *lamblia* infection caused a significant increase in antigen-specific serum antibodies of both isotypes, although the increases were generally greater for ENO over OCT (Fig 2A and 2B). Levels of ENO-specific IgG and IgA were significantly correlated (Fig 2C). In contrast, levels of IgG against ENO and OCT were only poorly correlated, since 58% (18/31) of the patients had IgG antibodies against ENO but not OCT, and an additional 13% (4/31) had antibodies against OCT but not ENO (Fig 2D). Only 3% (1/31) had IgG against both antigens, while 26% (8/31) did not have IgG against either antigen (Fig 2D). These findings confirm and expand prior findings [13] that both enzymes are immunogenic in humans after *G*. *lamblia* infection, although significant variability exists in the degree and isotypes of antibody induction between individuals.

### Protective capacity of ENO and OCT in murine models of giardiasis

**Fig 3:**
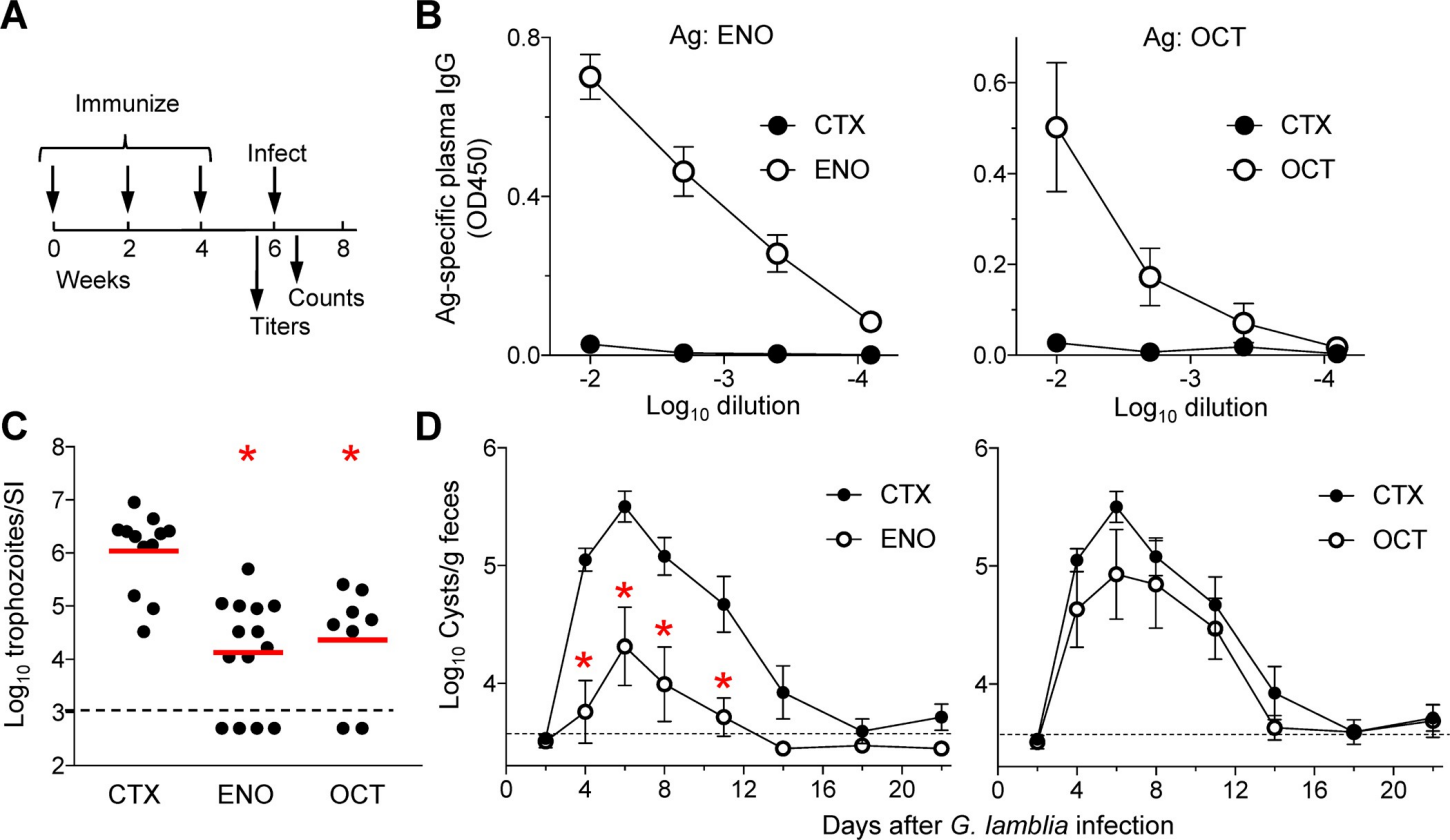
Immunogenicity and protective capacity of ENO and OCT in mice. A-C. C57BL/6 mice were immunized by three intranasal administrations two weeks apart with ENO or OCT, and cholera toxin (CTX) as an adjuvant. CTX toxin alone was used as a control. B. Mice were bled, and plasma IgG levels against the respective antigens (Ag) were determined by ELISA (means ± SE, n = 4 mice/group; open symbols, immunized mice; closed symbols, CTX-treated controls). C. Two weeks after the last immunization, mice were orally inoculated with *G*. *lamblia* GS/M trophozoites, and live trophozoites were enumerated in the small intestine (SI) after 5 days. Data from three separate experiments (i.e., separate inoculations on different days) were combined in the graph. Each dot represents an individual animal. The red lines represent geometric means, the dashed black line the detection limit of the assay. *P<0.05 vs CTX control as determined by Kruskal-Wallis test with Dunn’s *post hoc* test. D. Mice immunized with ENO (n = 5 mice) or OCT (n = 5 mice) and CTX-treated controls (n = 5 mice) were orally infected with *G*. *lamblia* GS/M trophozoites and fecal cyst output was determined at the indicated intervals. Data are shown as means and SE. *P<0.05 vs CTX control at the same time as determined by t-test.

Encouraged by the immunogenicity of the two enzyme antigens in >70% of giardiasis patients, we explored their protective capacity in a murine model of *G*. *lamblia* infection. Wild-type C57BL/6 mice were separately immunized with the purified recombinant proteins together with the universal mucosal adjuvant, cholera toxin, by intranasal administration (Fig 3A). Immunization induced strong IgG responses with a >1,000-fold increase in plasma titers against both antigens, demonstrating successful adaptive immune stimulation (Fig 3B). Immunized mice were then challenged by oral gavage with *G*. *lamblia* GS/M trophozoites, and parasite load in the small intestine was determined after five days, representing the peak of infection in controls. Immunization with both ENO and OCT caused a significant >100-fold reduction in trophozoite numbers compared to CTX-treated control mice (Fig 3C). A subset of mice (4/14, 29% for ENO and 2/8, 25% for OCT) had no detectable trophozoites (Fig 3C).

To explore the impact of immunization over the entire course of the infection, we performed fecal cyst counts at regular intervals. Consistent with the trophozoite findings, ENO immunization significantly reduced cyst excretion by 15- to 30-fold during the peak infection period between days 4 and 11 (Fig 3D, left). Furthermore, the duration of infection was shortened after ENO immunization, since fecal cysts were no longer detectable by day 14, while controls had detectable cysts for at least 18–22 days (Fig 3D, left). In contrast, fecal cyst output was only moderately and not significantly reduced in OCT-immunized mice challenged with *G*. *lamblia* GS/M (Fig 3D, right). Together, these data show that ENO is an effective antigen for vaccination against *G*. *lamblia* infection, while OCT appears to have a more limited protective capacity that only manifests itself at the level of intestinal trophozoite load but not fecal cyst shedding.

### Immunization with antigen combinations

**Fig 4:**
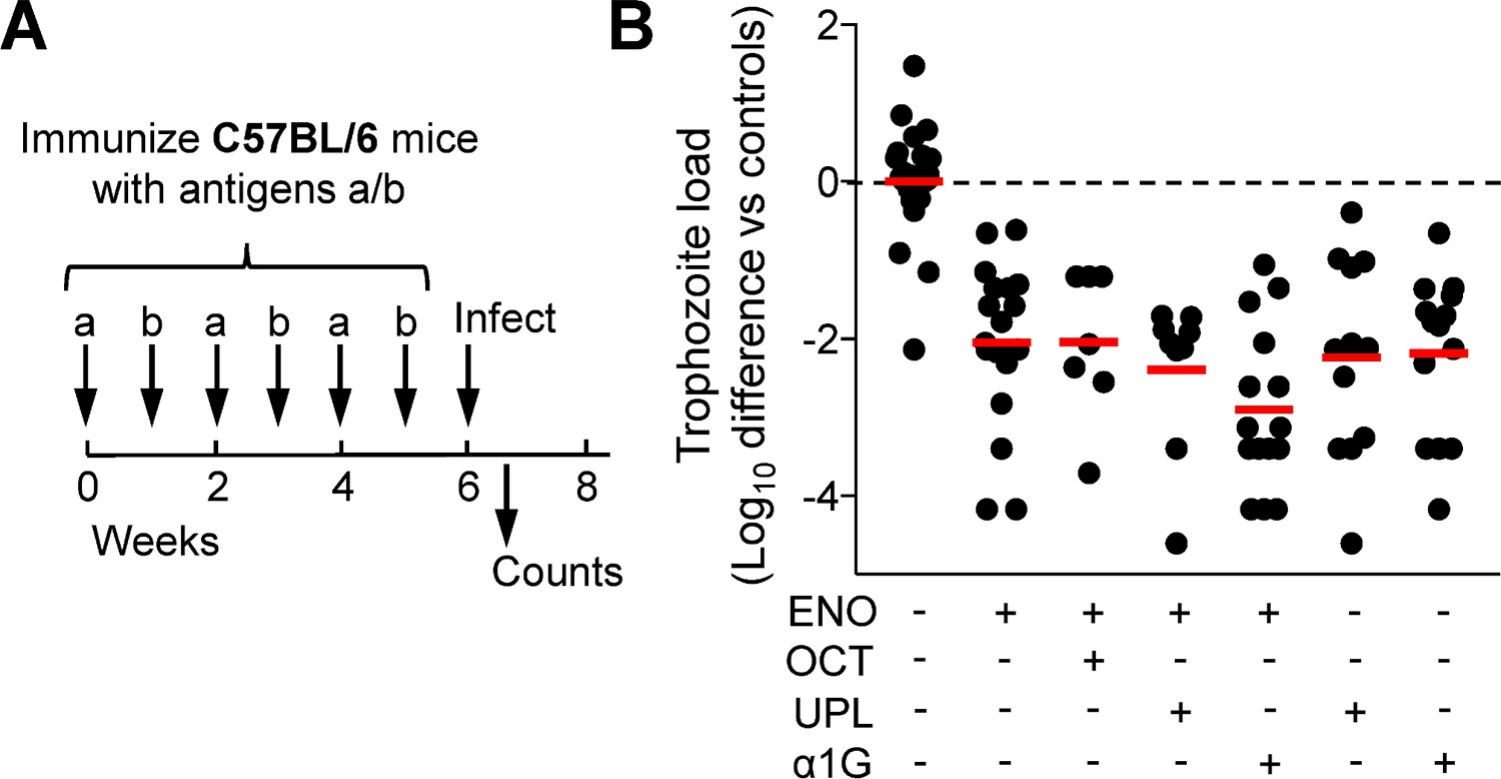
Effect of antigen combinations on protection against *G*. *lamblia*. A,B. C57BL/6 mice were immunized by alternating intranasal administrations of two antigens (a and b) on a weekly basis for a total of three doses for each, using cholera toxin as the adjuvant (A). Cholera toxin (CTX) alone was used as a control. One week after the last immunization, the C57BL/6 mice were orally inoculated with *G*. *lamblia* GS/M trophozoites, and live trophozoites were enumerated in the small intestine after 5 days. The graph in panel B shows the decrease in log_10_ of the trophozoite numbers in the small intestine relative to those of CTX controls for the indicated antigen combinations (ENO, α-enolase; OCT, ornithine carbamoyl transferase; α1G, α1-giardin; UPL, uridine phosphorylase-like protein-1). Each dot represents an individual animal. Data are compiled from five separate experiments, with a mean and SE of the log_10_ trophozoite load in the CTX controls of 5.78 ± 0.71 (range 4.83–6.41) on day 5. Red lines represent geometric means, the dashed black line shows the mean of the controls. All six immunized groups, which had received either one of three antigens (ENO, UPL, α1G) or two antigens (ENO+OCT, ENO+UPL, ENO+ α1G), were significantly protected (*P<0.05 by Kruskal-Wallis test with Dunn’s *post hoc* test), while none of these groups were significantly different from any other immunized group. Significances were omitted from the graph for clarity.

Given that a large proportion (22/31, 71%) of giardiasis patients showed immune responses to either ENO or OCT, but not both, we questioned whether immunization with both antigens might be more protective than either alone. To minimize any possible immune interference during co-immunization, we administered the antigens intranasally on an alternating schedule with one week recovery between individual immunizations (Fig 4A). Dual immunization with ENO and OCT did not lead to significantly better reduction in trophozoite load than immunization with ENO alone (Fig 4B). Furthermore, dual immunization with ENO and one of two other individually protective *G*. *lamblia* antigens that we had previously identified, α1-giardin and UPL-1 [26,27], did not significantly improve trophozoite reduction beyond what the individual antigens could confer (Fig 4B). However, we observed a modest trend towards greater reduction with ENO and α1-giardin compared to either antigen alone (Fig 4B). Together, these data suggest that combinations of two vaccine antigens generally do not provide additional immune protection beyond what can be achieved with the individual antigens.

### Cross-protection against divergent *G*. *lamblia* strains

**Fig 5:**
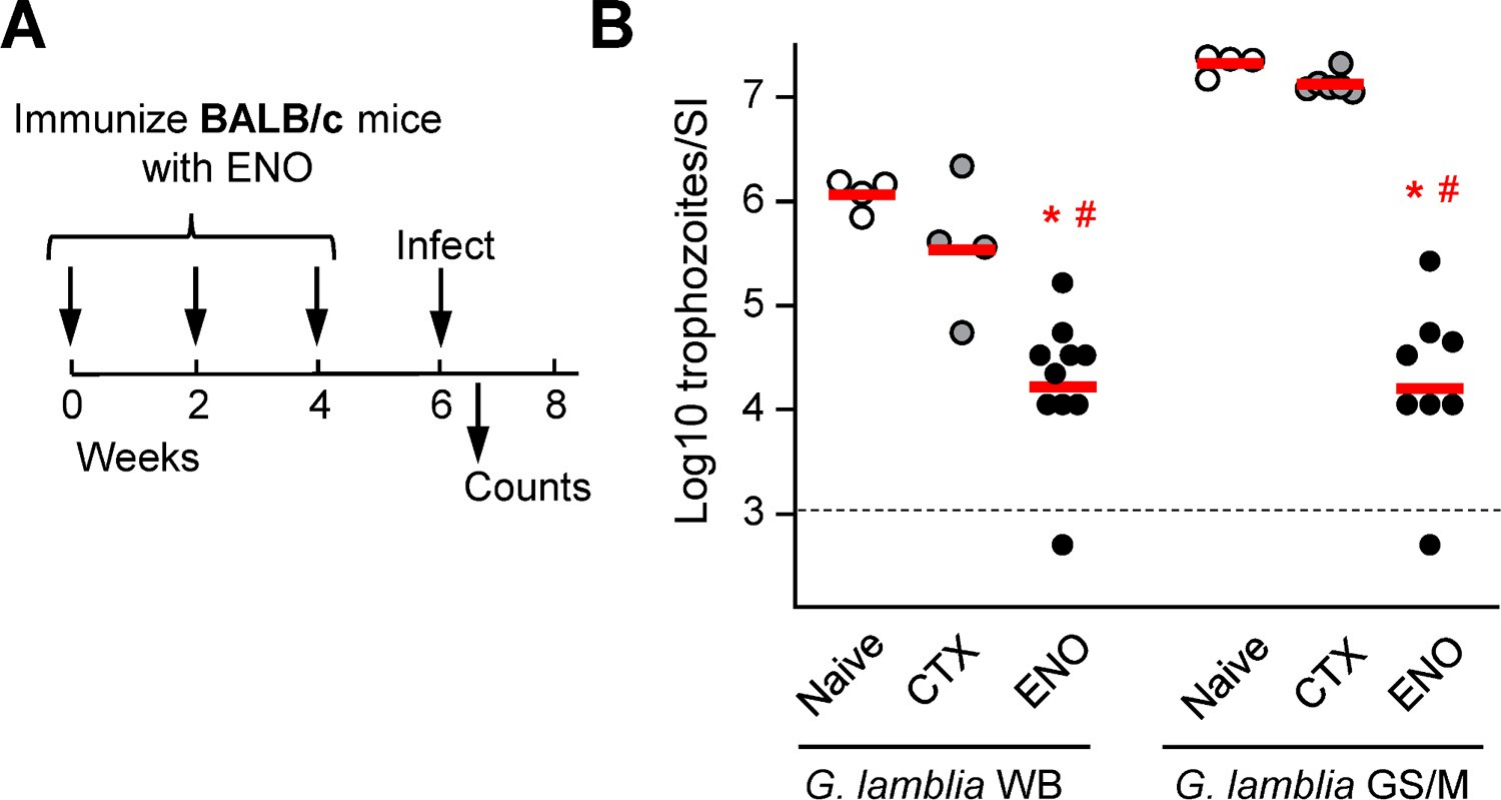
Cross-protection against divergent *G*. *lamblia* strains. BALB/c mice were immunized three times with ENO, using CTX as the adjuvant (A). CTX alone and naïve untreated mice were used as controls. Two weeks after the last immunization, the mice were orally inoculated with either *G*. *lamblia* WB or GS/M trophozoites, and live trophozoites were enumerated in the small intestine (SI) after 5 days (B). Each dot represents an individual animal. The red lines represent geometric means, the dashed black line the detection limit of the assay. Significance was evaluated by ANOVA, followed by a post-hoc Tukey test. *P<0.05 vs CTX controls, ^#^P<0.05 vs naive controls for the respective strain. CTX-treated and naïve mice were not significantly different for either *G*. *lamblia* strain.

To determine whether the target antigens, which are based on the genome sequence of the assemblage B strain GS/M, afford cross-protection against divergent *G*. *lamblia* stains, we focused on ENO because it had proven to confer more robust protection than OCT (see Fig 3). As a murine model, we employed BALB/c mice since they could be more consistently infected in our experiments than C57BL/6 mice with both *G*. *lamblia* strains, GS/M (assemblage B) and WB (assemblage A). Mice were immunized with ENO, challenged orally with *G*. *lamblia* GS/M trophozoites, and trophozoites in the small intestine were enumerated at the peak infection on day 5 (Fig 5A). Similar to the findings in C57BL/6 mice, ENO immunization led to a significant ~1,000 reduction in trophozoite numbers after GS/M infection relative to both CTX-treated and naïve controls (Fig 5B). Importantly, ENO also significantly reduced peak trophozoite load after WB infection (Fig 5B), although the infectious load was markedly (10-40-fold) lower in the CTX controls compared to GS/M and the reduction in ENO-immunized mice was more modest (~20-fold). Nonetheless, these data show that ENO can induce significant cross-protection against divergent *G*. *lamblia* strains, confirming that the degree of ENO sequence conservation between the assemblages is sufficient for effective immune responses against different strains.

### IgA is dispensable for ENO- and OCT-induced immune protection

**Fig 6:**
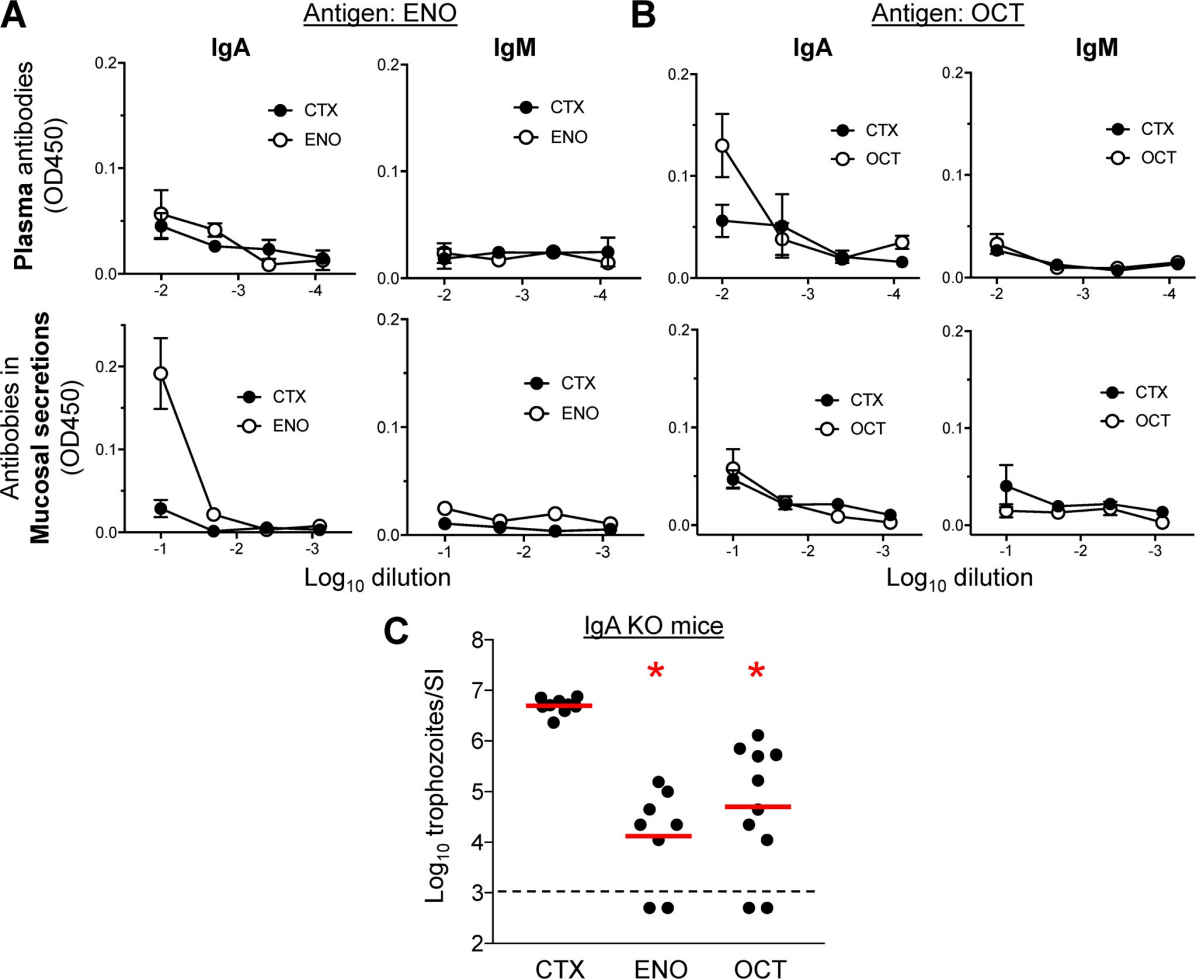
Role of IgA in ENO- and OCT-induced immune protection. A,B. C57BL/6 mice were immunized with ENO or OCT, and cholera toxin (CTX) as adjuvant, as detailed in Fig 3A. CTX alone was used as a control. Plasma and mucosal washes from the small intestine were obtained and tested by ELISA for IgA and IgM antibodies against the immunizing antigens. Data are means ± SE (n = 4 mice/group). C. IgA-deficient mice (KO) were immunized with ENO (n = 8 mice) or OCT (n = 10 mice), or treated with CTX alone as a control (n = 8 mice). Two weeks after the last immunization, mice were orally inoculated with *G*. *lamblia* GS/M trophozoites, and live trophozoites were enumerated in the small intestine (SI) after 5 days. Each dot represents an individual animal. Data are compiled from two separate experiments (inoculations). The red lines represent geometric means, the dashed black line the detection limit of the assay. *P<0.05 vs. CTX control as determined by Kruskal-Wallis test with Dunn’s *post hoc* test.

Mucosal IgA has been implicated in effective adaptive immunity against giardiasis [33], so we explored whether IgA is involved in the protection conferred by immunization with the target antigens. Intranasal immunization with ENO in the presence of cholera toxin as the adjuvant induced low levels of IgA in mucosal secretions from the small intestine but not in the plasma, while the opposite was seen for OCT, with low plasma IgA levels but no IgA in mucosal secretions (Fig 6A). Neither antigen induced specific IgM, which can compensate for IgA under some conditions, in plasma or mucosal secretions (Fig 6A). Furthermore, immunization with ENO or OCT in gene-targeted mice deficient in IgA production led to a 2–3 log_10_ reduction in peak *G*. *lamblia* numbers compared to CTX-treated IgA-deficient control mice (Fig 6B). This degree of trophozoite reduction was similar to what was observed in wild-type mice (see Fig 3C). Together, these data indicate that IgA is dispensable for ENO- and OCT-induced immune protection against *G*. *lamblia* infection.

### Immunization-induced IgG isotype distribution

**Fig 7:**
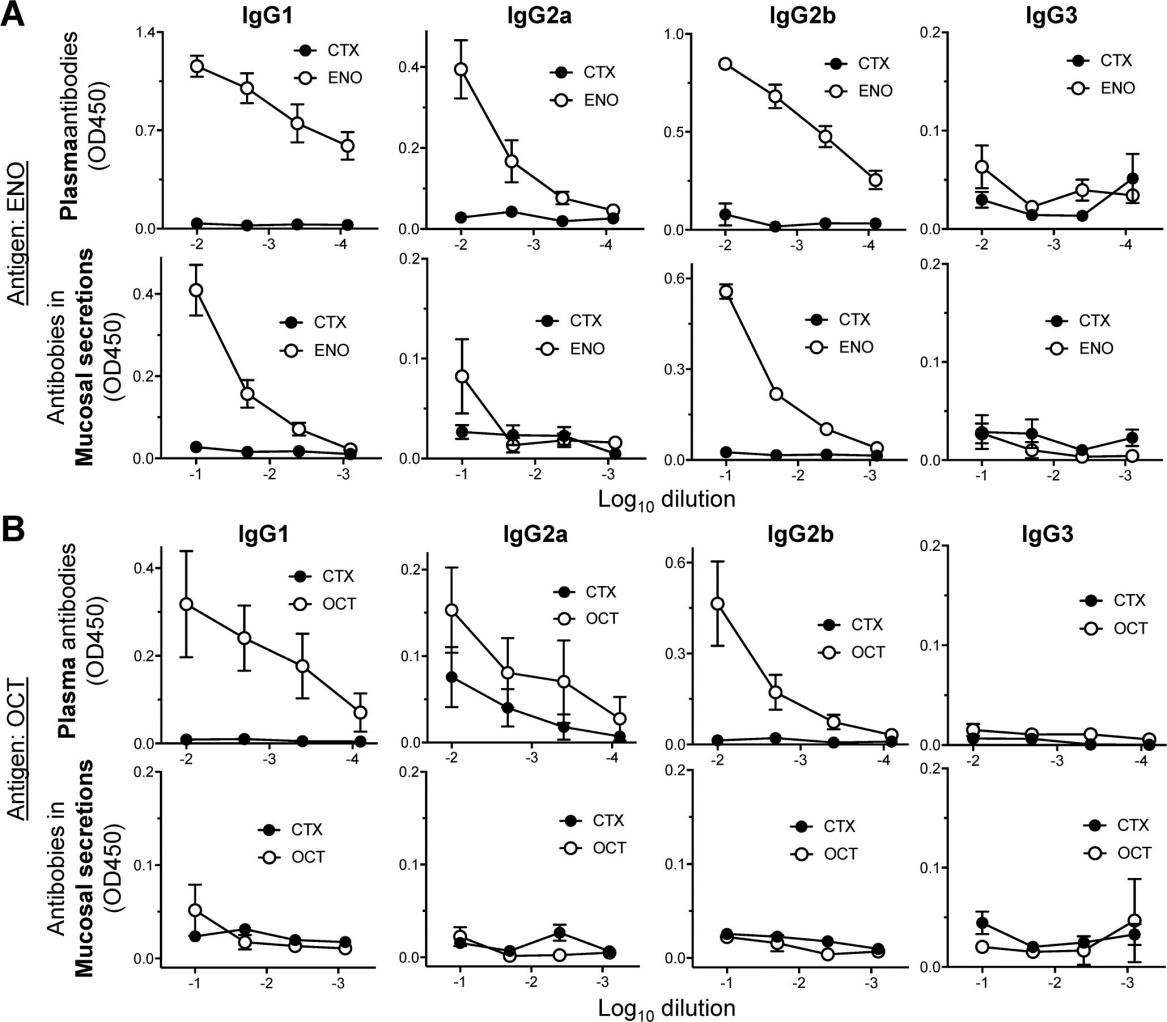
IgG isotypes after ENO and OCT immunization. C57BL/6 mice were immunized with ENO or OCT, and cholera toxin (CTX) as adjuvant, as detailed in Fig 3A. CTX alone was used as a control. Plasma and mucosal washes from the small intestine were obtained and tested by ELISA for the indicated IgG isotypes against the immunizing antigens. Data are means ± SE (n  = 4 mice/group).

Because IgA and IgM were not important in immunization-induced protection against infection, yet B cells were previously shown to play a critical role in clearance of giardiasis [33], we explored whether IgG isotypes might be involved. Determination of antigen-specific IgG antibodies after intranasal ENO immunization in the presence of CTX showed strong induction of IgG1 and IgG2b in plasma, which was paralleled by significant induction of these isotypes in mucosal secretions, whereas IgG2a was only modestly induced in plasma and not found in mucosal secretions, and IgG3 was not induced in plasma or mucosal secretions (Fig 7A). By comparison, immunization with OCT also induced, albeit more modestly, plasma IgG1 and IgG2b, but these isotypes were not seen in mucosal secretions (Fig 7B). OCT did not induce any IgG2a or IgG3 in plasma or mucosal secretions (Fig 7B). These results suggest that ENO immunization induces strong IgG1 and IgG2b responses detectable in both plasma and mucosal secretions, while OCT is a weaker inducer of these responses in plasma and not at all in mucosal secretions, perhaps providing a partial explanation for the differences in the reduction of trophozoite vs cyst numbers that was seen for ENO and OCT (see Fig 3).

### Intestinal cytokine responses after immunization and *G*. *lamblia* challenge

**Fig 8:**
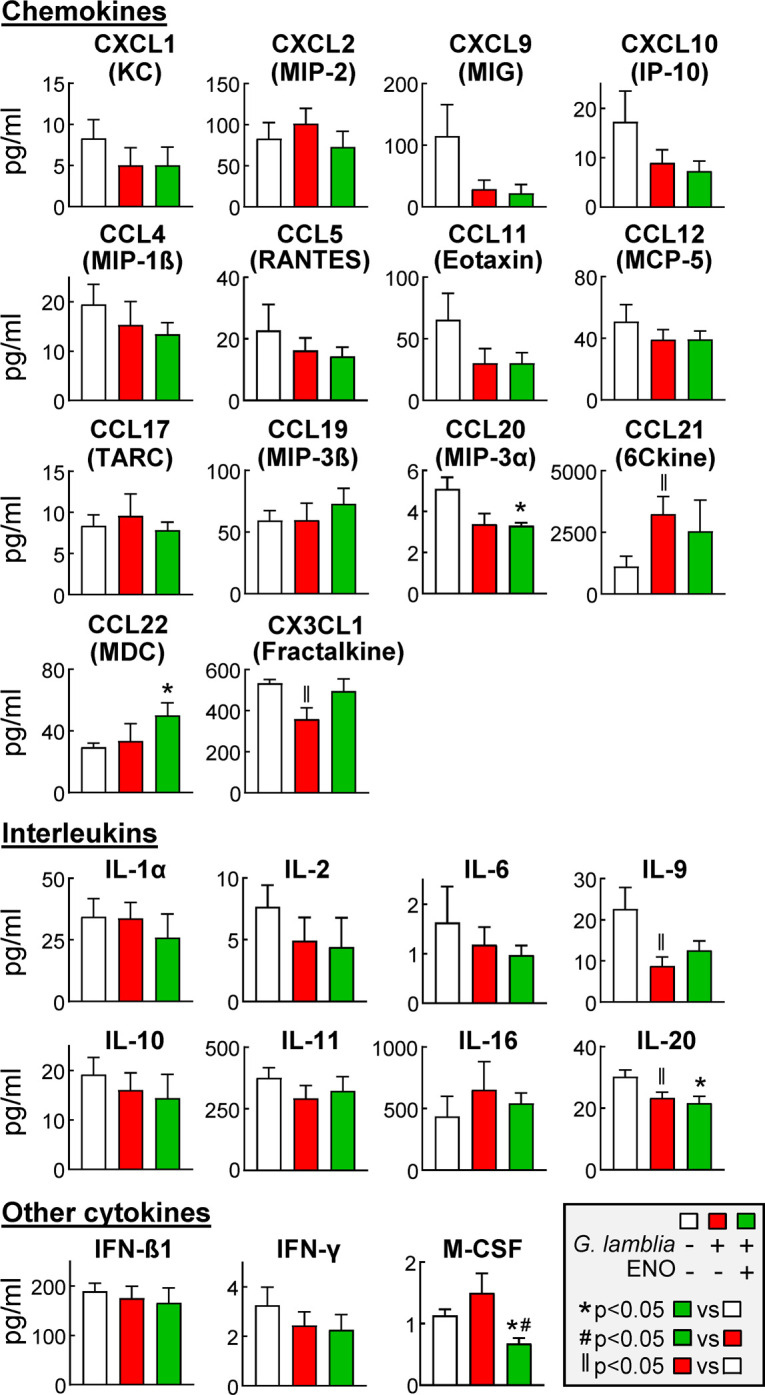
Intestinal cytokine expression after *G*. *lamblia* infection of ENO-immunized mice. C57BL/6 mice were immunized with ENO and cholera toxin (CTX) as adjuvant, as detailed in Fig 3. Treatment with CTX alone and naïve mice were as controls. ENO-immunized and CTX-only mice were orally infected with *G*. *lamblia* GS/M, while naïve mice were left uninfected. After 5 days, extracts were prepared from the mid-small intestine and levels of the indicated cytokines were assayed by multiplex immunoassay. Data are mean ± SE (n = 6 mice/group). Significances were determined by t-test.

For further exploration of potential mechanisms of immunization-induced protection against *G*. *lamblia* infection, we examined intestinal cytokine responses in mice immunized with the more potent of the two target antigens, ENO, or treated only with CTX as a control, and challenged with *G*. *lamblia*. Naïve mice not immunized or infected were used as an additional control. Intestinal extracts were prepared at the peak of infection (day 5) and tested by multiplex immunoassays for the levels of 44 immune cytokines and other immune-related proteins. Levels of most cytokines were similar in the three cohorts for the 25 cytokines with detectable protein expression (Fig 8), while the remaining 19 cytokines (G-CSF, GM-CSF, IL-1β, IL-3, IL-4, IL-5, IL-7, IL-12p40, IL-12p70, IL-13, IL-15, IL-17, LIF, LIX, CCL2/MCP-1, CCL3/MIP-1α, TIMP-1, TNFα, and VEGF-A) were below the detection sensitivity of the assay. A slight trend towards lower levels in the ENO-immunized infected mice was observed for several cytokines (e.g. CXCL1, CXCL9, CXCL10, CCL11, CCL20, IL-2, IL-9, IL-20, M-CSF), albeit the decreases reached significance relative to naïve mice only for CCL20, IL-20, and M-CSF, and relative to infected CTX controls only for M-CSF (Fig 8). The only cytokine that was found to be increased in ENO-immunized infected mice was CCL22, showing a modest but significant ~2-fold increase relative to naïve controls and a ~1.5-fold increase compared to infected CTX controls. Overall, these results do not reveal a strong candidate cytokine whose altered intestinal production might explain the ENO-induced immune protection, although CCL22 shows some promise due to its relatively unique, albeit modest, intestinal induction in ENO-immunized mice.

### Immune cell profiles in small intestine and mesenteric lymph nodes after *G*. *lamblia* infection of ENO-immunized mice

**Fig 9:**
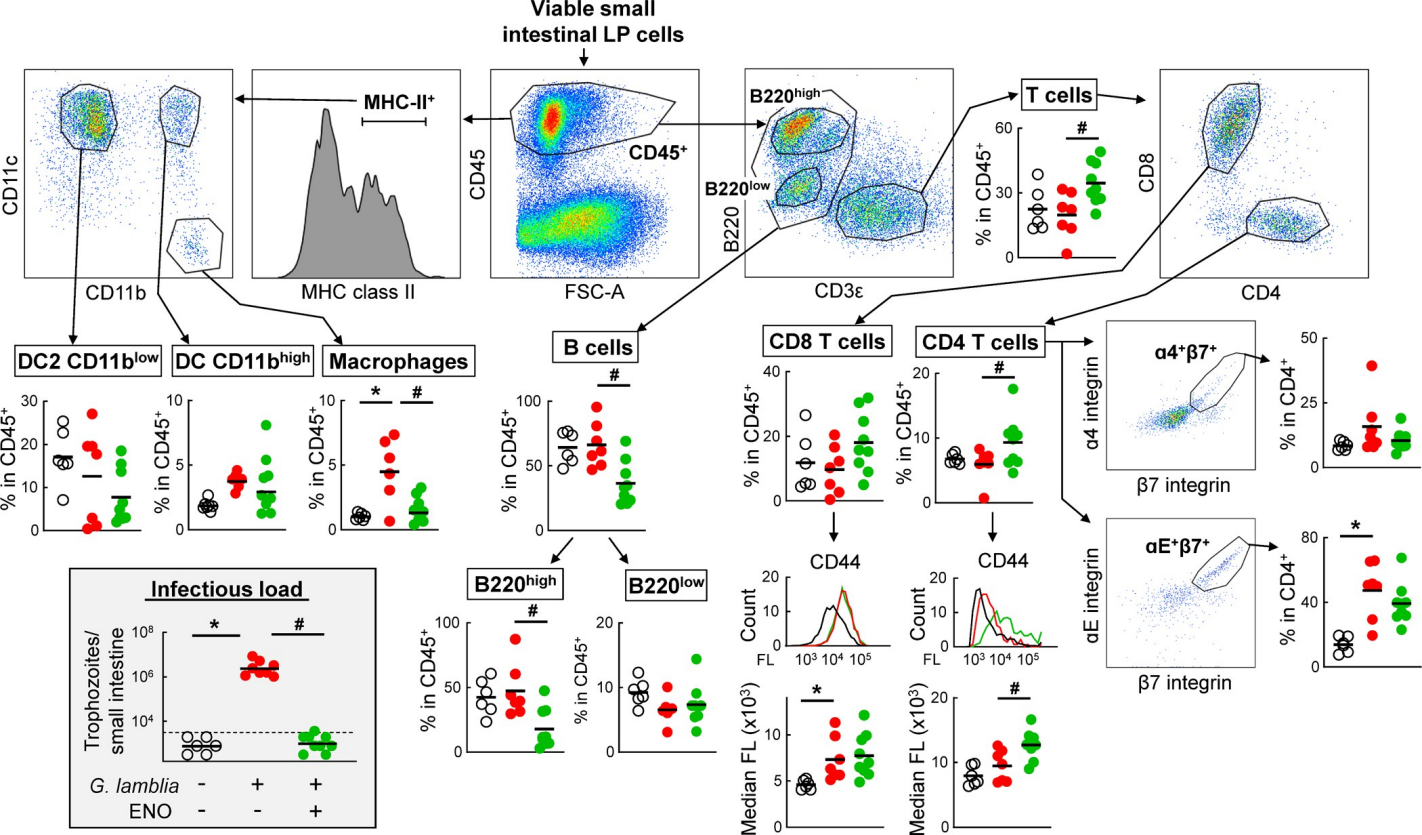
Spectral flow cytometric analysis of intestinal immune cell populations. C57BL/6 mice were immunized with ENO and CTX (green symbols), as detailed in Fig 3. Treatment with CTX alone (red symbols) and untreated naive mice (open black symbols) were used as controls. ENO-immunized and CTX-only mice were orally infected with *G*. *lamblia* GS/M, while naïve mice were left uninfected. After 5 days, single-cell suspensions were prepared from the small intestine. Cells were stained with a viability dye and a cocktail of antibodies against the indicated markers and analyzed by spectral flow cytometry. Dot plots depict representative examples for the gating strategies, while the graphs show the results for individual animals, with means indicated by horizontal bars. Data are compiled from two separate experiments. Significances were calculated by t-test (*p<0.05 for infected CTX controls vs naïve mice; #p<0.05 for infected ENO-immunized mice vs infected CTX controls). The graph in the gray-shaded area shows trophozoite counts obtained from the same cohorts.

Since the cytokine assays did not yield clear insights into potential mechanism of immunization-induced protection, we next asked whether changes in the composition of the major immune cell types in the small intestine, the key effector site of the host response to *Giardia*, may provide some clues. ENO-immunized mice and CTX-treated controls were infected with *G*. *lamblia* GS/M, with naïve mice as an additional control. At the peak of infection (day 5), single cell suspensions were prepared from the small intestine, and analyzed for a broad range of immune cell markers by spectral flow cytometry. A significant increase in T cells was observed in ENO-immunized infected mice compared to infected CTX controls and naïve mice (Fig 9). The difference was largely due an increase in CD4 T cells. In parallel, the CD4 T cells in ENO-immunized infected mice displayed significantly increased activation, as determined by CD44 expression, and had a modest increase in the proportion of αEβ7-positive cells, although that was also seen in infected CTX controls compared to naïve mice. CD8 T cells were not notably increased in numbers, although they showed signs of increased activation (CD44 expression) in infected mice regardless of immunization status (Fig 9). B cells were decreased in ENO-immunized infected mice compared to both control groups, which was mostly explained by a decrease in B220^high^ cells, representing immature B cells. Dendritic cells, both CD11b^low^ and CD11b^high^ populations, showed substantial numeric variability but no significant trends between the cohorts (Fig 9). Macrophages (defined here as MHC II^+^, CD11b^+^, CD11c^-^ cells) were significantly increased in infected CTX controls, but not in the ENO-immunized infected group (Fig 9). Together, these findings most prominently suggest an increase in the number and activation state of CD4 T cells in the lamina propria of the small intestine of ENO-immunized mice upon *G*. *lamblia* infection.

**Fig 10:**
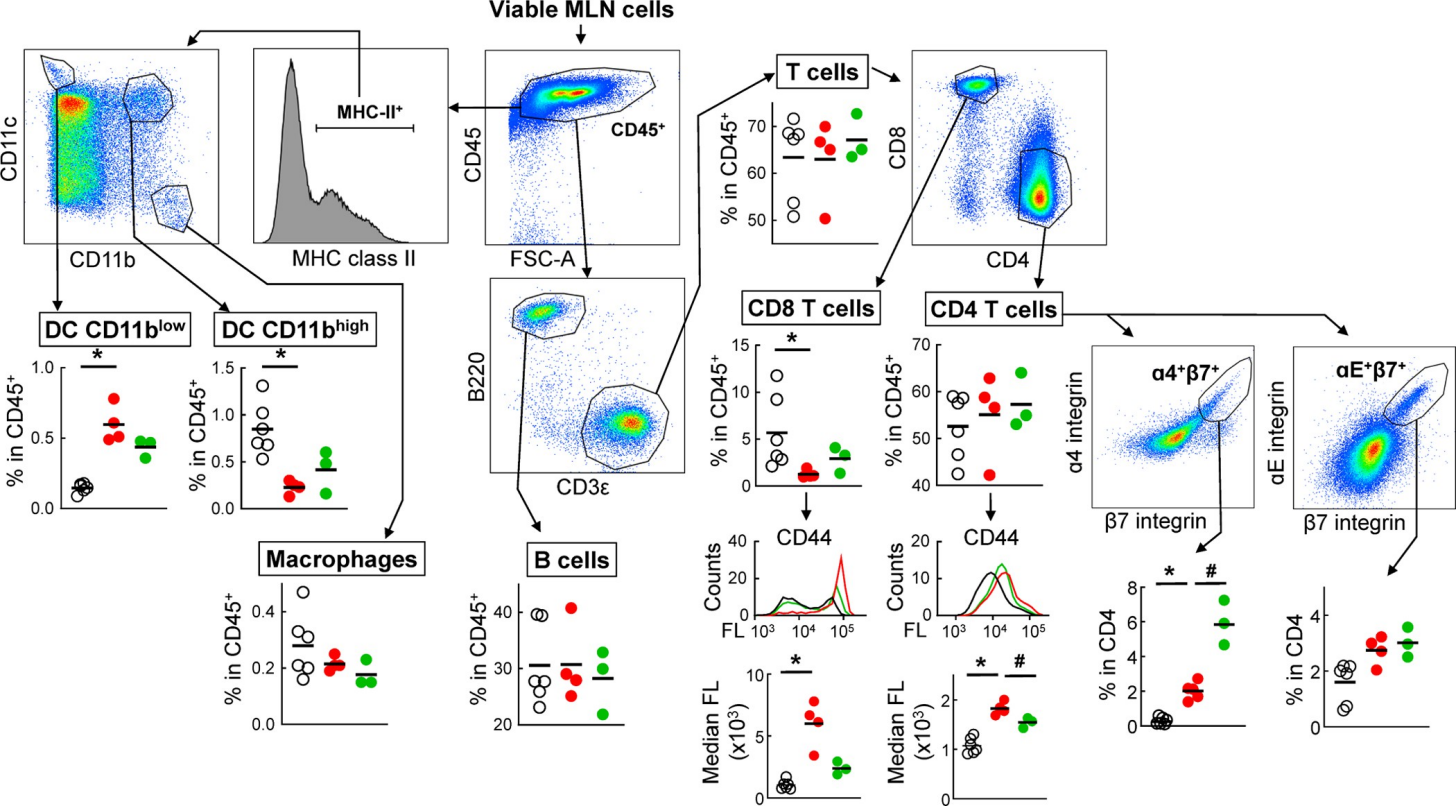
Spectral flow cytometric analysis of immune cell populations in the mesenteric lymph nodes. C57BL/6 mice were immunized with ENO and CTX (green symbols), as detailed in Fig 3. Treatment with CTX alone (red symbols) and untreated naive mice (open black symbols) were used as controls. ENO-immunized and CTX-only mice were orally infected with *G*. *lamblia* GS/M, while naïve mice were left uninfected. After 5 days, single-cell suspensions were prepared from the mesenteric lymph nodes. Cells were stained with a viability dye and a cocktail of antibodies against the indicated markers and analyzed by spectral flow cytometry. Dot plots depict representative examples for the gating strategies, while the graphs provide the results for individual animals, with means indicated by horizontal bars. Significances were calculated by t-test (*p<0.05 for infected CXTX controls vs naïve mice; #p<0.05 for infected ENO-immunized mice vs infected CTX controls).

Given the interconnections of the common mucosal immune system, we also investigated immune cell composition in the mesenteric lymph nodes (MLN) as the primary draining lymph nodes and waystation for immune cell trafficking to and from the intestine. Contrary to the small intestine, neither total T cells nor CD4 T cells were significantly altered after *G*. *lamblia* infection of ENO-immunized mice, although the cells showed increased activation as reflected by CD44 expression (Fig 10). Notably, the proportion of CD4 T cells expressing the intestinal homing receptor, α4β7, was significantly increased relative to infected CTX controls and naïve mice, whereas cells expressing αEβ7 were not significantly changed. CD8 T cells were decreased in infected CTX controls and to a lesser extent in infected ENO-immunized mice, which was paralleled by marked activation of CD8 T cells, particularly in the infected CTX controls (Fig 10). Dendritic cells showed a subset-specific response, with CD11b^low^ cells increased in infected CTX controls, and to a lesser extent in infected ENO-immunized mice, while CD11b^high^ cells were decreased (Fig 10). B cells and macrophages were not significantly altered in the MLN in any of the groups. Overall, the most significant MLN finding was the increase in α4β7-expressing CD4 T cells in ENO-immunized mice, as these cells are destined to return to the intestine and exert effector functions.

### CD4 T cells are critical for ENO- and OCT-induced protection

**Fig 11:**
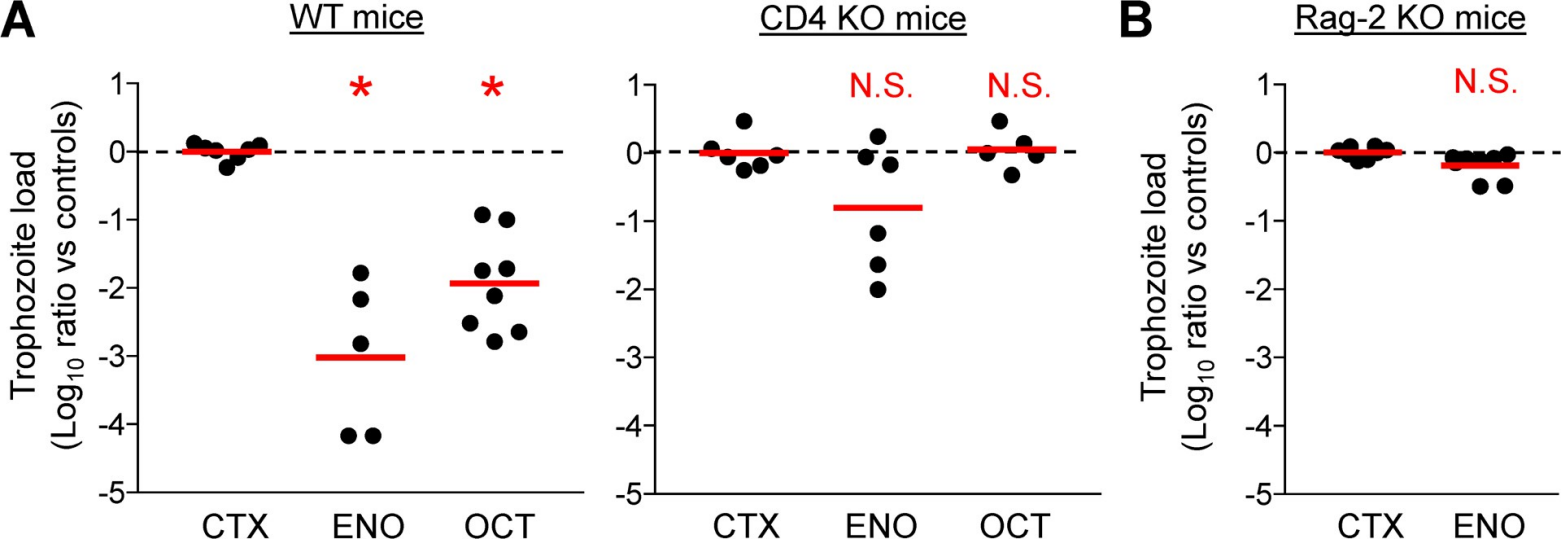
CD4 T cell dependence of vaccine-induced protection against *G*. *lamblia*. CD4-deficient (A, KO) and C57BL/6 mice (A, WT) mice, and Rag-2-deficient (B, KO) mice, were immunized intranasally with the indicated antigens and cholera toxin (CTX) as an adjuvant. CTX alone was used as a control. Two weeks after the last immunization, mice were orally inoculated with *G*. *lamblia* GS/M trophozoites, and live trophozoites were enumerated in the small intestine after 5 days. The graphs show the decrease in log_10_ of the trophozoite numbers in the small intestine relative to those of controls for individual mice. Data are compiled from two separate experiments, with mean day 5 log_10_ trophozoite loads in the CTX controls of 6.87 and 7.31 (A, WT mice), 6.53 and 5.06 (A, CD4 KO), and 7.20 and 6.99 (B, Rag-2 KO). The red lines represent geometric means and the dotted lines represent the trophozoite load in controls. *P<0.05 vs. CTX controls as determined by Kruskal-Wallis test with Dunn’s *post hoc* test (A); N.S., not significant.

Because of the observed increase and activation of CD4 T cells in the small intestine of ENO-immunized mice, combined with the increase in the proportion of these cells expressing the intestinal homing receptor, α4β7, in the MLN, we explored whether the cells are critical for immunization-induced protection against *G*. *lamblia* infection. CD4-deficient mice were intranasally immunized with ENO, as well as OCT, together with cholera toxin, and challenged two weeks later with *G*. *lamblia* GS/M trophozoites by oral gavage. Immunization with either antigen failed to induce significant reduction in trophozoite numbers relative to CTX-treated CD4-deficient control mice, although ENO immunization appeared to modestly reduce trophozoite numbers in a subset of mice (Fig 11A). Because ENO immunization appeared to have a partial, albeit not significant, protective effect, we further explored whether any T and B cells are involved. Immunization of Rag 2-deficient mice, which lack almost all T and B cells, caused no reduction in trophozoites numbers in any of the mice compared to CTX-treated Rag-2-deficient control mice (Fig 11B). These data show that CD4 T cell-mediated immunity is critical for protection induced by immunization of mice with ENO, as well as OCT.

## Discussion

Vaccination is one of the most successful strategies to prevent a host of infectious diseases, yet this promise remains unfilled for giardiasis. The underlying reasons are manifold, relating to biological, medical and economic challenges. Foundational to any successful vaccine development is the identification and validation of suitable antigens. For giardiasis, a number of antigen candidates have been identified, including VSPs [21], α1-giardin [27], and additional proteins found on the surface of the trophozoites [26]. Other proteins such as binding immunoglobulin protein [34] and excretory-secretory products [35] have uncertain localizations or functions, but could also be useful for vaccine design. The current study adds to the repertoire of vaccine antigen candidates by confirming that the two metabolic enzymes, ENO and OCT, are immunogenic in mice and humans and demonstrating that they confer protection against *G*. *lamblia* infection in a murine model. Their excellent conservation across *G*. *lamblia* assemblages and our observation of cross-protection between divergent *G*. *lamblia* strains, combined with the lack of closely related human proteins, make them valuable new candidates for the development of a vaccine against giardiasis.

ENO catalyzes the reversible interconversion of 2-phosphoglycerate and phosphoenolpyruvate in the glycolytic pathway of both prokaryotes and eukaryotes [36]. In addition, ENO has virulence-related functions in *Giardia* [37] and other eukaryotic pathogens [38]. OCT mediates the production of citrulline from ornithine and carbamoylphosphate, which plays an important role in arginine metabolism and ATP generation in *G*. *lamblia* [39]. Both enzymes are located in the cytoplasm where the metabolic reactions occur that they catalyze [40,41]. This location poses the question how these giardial proteins can be immunogenic as they would not be expected to have contact with the mucosal immune system unless trophozoites undergo cell lysis, but little evidence exists that this occurs in vivo. However, despite the localization of ENO and OCT in the cytoplasm and at the inside of the plasma membrane in trophozoites [42], the enzymes are upregulated and released in response to interaction with intestinal epithelial cells in the apparent absence of lytic cell death [42–44]. Similarly, ENO, which has cytoplasmic metabolic functions in *Leishmania* spp., can be secreted by those organisms [38]. Although the underlying mechanisms are not well understood in *Giardia* [44,45], the host cell-induced enzyme release from the parasite could explain how antigen-specific immune responses against ENO and OCT might be activated during *G*. *lamblia* infection.

As secreted proteins, the two newly validated vaccine antigens are likely to be disconnected from the parasite during infection, questioning how they might mediate anti-parasitic host defense. For surface proteins, host effectors such as secreted IgA can directly bind to trophozoites and entangle them [14], thereby preventing them from normal attachment to the epithelial surface through their ventral disc [46]. Such a mechanism would presumably not be relevant for secreted proteins since they are detached from trophozoites, so an antibody response would not directly impact the trophozoites. Consistent with this notion, deficiency in secretory IgA, which could directly bind to cognate targets on the parasite surface, did not interfere with the immune protection conferred by ENO or OCT. Instead, we could speculate that such secreted antigens serve as broad indicators of *G*. *lamblia* infection in the host. In this scenario, their secretion by the parasite and subsequent immune detection can activate non-specific luminal defenses such as intestinal hypermotility [47,48], mucus release [49] or mast cell-activated diarrhea [50], independent of their physical association with the parasite.

Combinations of antigens have the potential to stimulate more effective antimicrobial immunity than single antigens since they might lead to greater cumulative immune activation and could potentially engage different immune effectors. However, the current study provides little support for this concept, since dual antigen combinations did not result in significantly greater protection than the individual antigens. Our tests involved various combinations of secreted [13,42] and membrane-associated antigens [26], further strengthening the conclusion. However, our study has limitations since it cannot exclude the possibility that combination of the right antigens could yield vaccine candidates with improved efficacy. For example, we saw a trend towards greater protection when ENO was combined with α1-giardin, so broader screens of combinations of conserved antigens and optimized immunization protocols may well yield better combination vaccines in future studies.

Adjuvants and suitable administration routes are critical for effective induction of mucosal immunity, particularly for pathogens such as *G*. *lamblia* that are restricted to the surface and lumen of the intestinal tract. For the present study, we used the prototype adjuvant cholera toxin and the commonly employed intranasal route of immunization, since the focus was on exploring the protective capacity of the targeted antigens, not on optimizing adjuvant strategies. Cholera toxin activates a broad range of immune effectors, including induction of secretory IgA by priming dendritic cells to induce IgA switching in B cells in a cyclic AMP- and TLR-dependent manner that is independent of T cells, IL-12 and IL-17A [51,52]. The IgA response was borderline in our system and clearly not important for protection, but we also observed strong IgG1 and IgG2b responses to the immunizing antigens, consistent with prior studies on cholera toxin adjuvanticity upon intranasal administration [53]. IgG isotypes can reach the intestinal lumen and mediate effector functions in that location [54], which may provide an explanation for the greater effectiveness of ENO in reducing cysts output compared to OCT, which neither induced IgG in mucosal secretions not attenuated cyst excretion.

Despite its utility in preclinical animal studies, cholera toxin and related enterotoxins are not approved for human use due to toxicity concerns [55,56], although mutant forms of enterotoxins have shown promise as mucosal adjuvants [57]. Other alternatives exist. For example, in a prior study, we had used antigens expressed from live attenuated *Salmonella*-based vectors without additional adjuvants, although those studies showed less efficacy, prompting the switch to the current approach [27]. Improvements in live vaccine vectors [58] may well justify reexamination of the best protective antigens identified here and in prior work [26,27] with vectored vaccines in murine giardiasis models. Furthermore, future development of a human vaccine for giardiasis will require optimization of adjuvants and delivery strategies, since the immunization approaches effective in mice can probably not be directly transferred to humans. For example, approved human vaccine adjuvants such as alum and its combinations with other immune stimulators or oil-in-water emulsions (e.g. MF59 or AS03) could be tested in different administration regimens with the newly identified protective antigens in preclinical murine models before consideration of human studies [59].

Mice deficient in T and B cells, or only lacking CD4 T cells, were not protected by immunization with ENO or OCT, which indicates that intact adaptive immunity is required for protection and that CD4 T cells play a primary role in this process. This observation is consistent with prior work on the central importance of CD4 T cells in controlling *G*. *lamblia* infection in mice [60]. In humans, the evidence for a role of CD4 T cells in giardiasis is not as clear, although several clinical studies suggest an association of decreased CD4 T cell numbers with increased *Giardia* prevalence and greater risk of symptomatic infection [61,62]. The mechanisms by which CD4 T cells mediate vaccine-induced protection remain to be elucidated, but their known role in regulating mucosal IgA responses [63] is not likely to be relevant. Nonetheless, our findings suggest that antigen-specific CD4 T cell responses could serve as a surrogate marker of effective immunization in future preclinical and clinical studies.

Our studies have limitations in that we could not assess markers of clinical disease in the murine models, since mice do not develop diarrhea upon *G*. *lamblia* infection. In future studies, it will be of interest to determine whether other host responses to infection, such as the reported changes in bile acid or lipid metabolism [64], would be attenuated by prior immunization with ENO or OCT. Furthermore, such studies could also assess the relationship between trophozoite load, which we used as a surrogate for disease severity, fecal cysts counts, and clinical disease manifestations. Finally, the observed differential impact of ENO vs OCT immunization on reduction of cysts excretion could have implications for the potential of community protection with different vaccine antigens, since humans are a major reservoir for the spread of giardiasis.
